# <i>CYLD</i> GeneticTesting for Brooke-Spiegler Syndrome, Familial Cylindromatosis and Multiple Familial Trichoepitheliomas

**DOI:** 10.1371/currents.eogt.45c4e63dd43d62e12228cc5264d6a0db

**Published:** 2015-02-19

**Authors:** Anna Dubois, Valerie Wilson, David Bourn, Neil Rajan

**Affiliations:** Department of Dermatology, Royal Victoria Infirmary, Newcastle upon Tyne, UK; Molecular Genetics Laboratory, Northern Genetics Service, Institute of Genetic Medicine, Newcastle upon Tyne, UK; Molecular Genetics Laboratory, Northern Genetics Service, Institute of Genetic Medicine, Newcastle upon Tyne, UK; Department of Dermatology, Royal Victoria Infirmary, Newcastle upon Tyne, UK; Institute of Genetic Medicine, Newcastle University, Newcastle upon Tyne, UK

## Abstract

The clinical presentation of multiple, rare, skin appendage tumours called cylindromas has been attributed to germline mutations in the tumour suppressor gene <i>CYLD</i> (OMIM 605018). Brooke-Spiegler Syndrome (BSS), familial cylindromatosis (FC) and multiple familial trichoepitheliomas (MFT) (OMIM #605041, #132700, #601606 respectively) differ due to the types of other skin appendage tumour seen together with cylindroma, such as spiradenoma and trichoepithelioma. Previously thought to be separate entities, they are now viewed as allelic variants with overlapping phenotypes, supported by mutation analysis of <i>CYLD</i> . The conditions display autosomal dominant inheritance and affected individuals develop multiple benign skin tumours most commonly on the head and neck. <i>CYLD</i> testing can be performed using PCR and Sanger sequencing for patients with: 1. Multiple cylindromas, spiradenomas or trichoepitheliomas. 2. A single cylindroma, spiradenoma or trichoepithelioma and an affected first-degree relative with any of these tumours. 3. An asymptomatic family member at 50% risk with a known mutation in the family.

## Clinical Phenotype

BSS, FC and MFT arise from heterozygous mutations in the *CYLD* gene^1^. Cylindromas are benign appendageal tumours occurring mainly on the scalp, but can occur on any hair bearing skin. The lesions typically present as painless, smooth pink nodules, which may be either solitary or clustered together. They are slow-growing and vary in size from a few millimetres to over six centimetres.

**Cylindroma:**
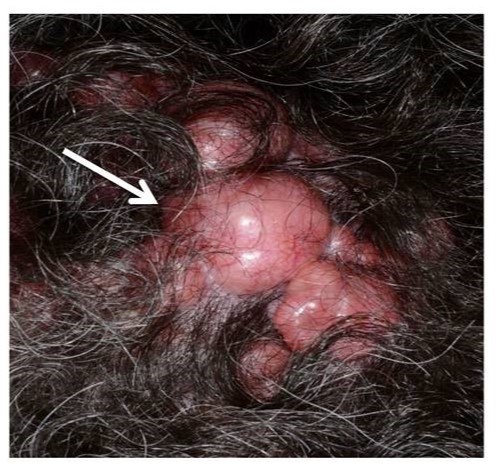
Several well-circumscribed, pink, nodular cylindromas with arborizing blood vessels on the surface, occurring on the scalp of a patient with BSS.

Spiradenomas often occur in conjunction with cylindromas in BSS, but tend to be painful rather than painless. They can present as a dermal nodule, often with a blue/black appearance.

**Spiradenoma:**
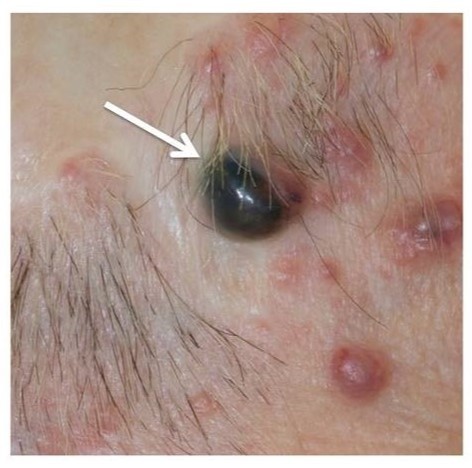
Nodular lesion with characteristic blue/black appearance.

Definitive diagnosis of cylindromas and spiradenomas requires histopathological examination of an excised lesion. Histopathologically, cylindromas consist of nests of basaloid cells arranged into nodules that resemble a cylinder in cross section. These form an irregular pattern, often a likened to a jigsaw.

**Histology of a cylindroma:**
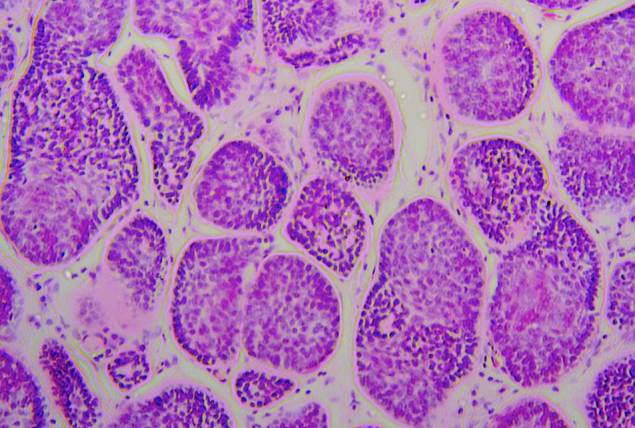
The typical appearance of a cylindroma at low power (10x), consisting of well-defined nests of basaloid cells separated by an eosinophilic basement membrane.

These tumours are rare in the general population, and a histology result should raise the suspicion of a germline *CYLD* mutation in a young person.

A diagnosis of FC or MFT is prompted by the dominant tumour type being cylindroma or trichoepithelioma respectively. BSS presents with a variety of skin appendage tumours including cylindromas, spiradenomas and trichoepitheliomas. The distinction made between the different conditions hence depends on the combination of tumour types identified. These divisions are not thought to be useful in the clinic in terms of prognostic information or counselling patients, and hence the term *CYLD* cutaneous syndrome has been proposed^2^ . The timing of tumour onset is usually in adolescence, but can occur from after adrenarche in childhood, up to the 4^th^ decade. The skin appendage tumours have a predilection for the face and scalp, although also affect the trunk, most commonly in hair-bearing areas^2^ .

The morbidity associated with skin appendage tumours in these conditions can be substantial. The tumours are disfiguring, can be painful and occur in multiple sites. Affected individuals face repeated episodes of surgery to remove lesions, and in some cases this may culminate in removal of the entire scalp^3^ . Patients may request surgery when tumours are painful, ulcerated, bleeding, unsightly or causing sexual dysfunction^4^ . In affected families, genetic testing allows individuals to ascertain their own risk and to use the information for family planning purposes.

## Test Description

Blood in EDTA is the preferred tissue but buccal swabs, mouthwash samples or solid tissue can also be tested. The testing protocol involves PCR amplification of exons 4-20 of *CYLD* in a total of 18 amplicons. In addition to the exonic sequence at least 10bp of flanking intronic sequence is captured at the 5’ and 3’ end of each exon. Following PCR amplification each amplicon is sequenced by bi-directional fluorescent Sanger sequencing and the data analysed using the current version of Mutation Surveyor™ software.****

## Public Health Importance

The estimated prevalence of *CYLD* mutations in the UK population is 1:100000, although this is difficult to accurately determine. The penetrance in terms of tumour development in those with a mutation in *CYLD* is estimated to be close to 100%. Most cases of BSS, FC and MFT result from autosomal dominant inheritance, although sporadic cases can also occur.

A genetic test result allows an individual to make decisions relating to family planning. It can also be useful to inform treatment planning. Patients with a *CYLD* mutation may be offered the opportunity to enrol into clinical trials. *CYLD *encodes a deubiquitinating enzyme that negatively regulates the nuclear factor-kappa beta pathway. A clinical trial using topical salicylic acid was not promising^5^ . More recently gene expression profiling of tumours in patients with germline *CYLD* mutations have shown dysregulated tropomyosin kinase (TRK) signalling, and treatment with lestaurtinib, a TRK inhibitor reduced growth of cells established from *CYLD *mutant tumours in vitro^6^ .

## Published reviews, recommendations and guidelines

Systematic evidence reviews: None identified

Recommendations by independent groups: UK GTN – Gene dossier

Guidelines by professional groups: None identified

## Evidence Overview

**Analytic validity:**

The testing methodology involves bi-directional fluorescent DNA sequencing of all coding exons of CYLD (exons 4-20, exonic sequence plus at least 10bp at the 5’ and 3’ of each intron). This method would be expected to detect &gt;99% of mutations within the coding region/splice sites, excluding duplications and large deletions. Large deletions have been reported to affect a minority of patients that are mutation negative following Sanger sequencing.^7^ cDNA testing is also carried out where appropriate. The generic techniques are well established in the CPA-accredited Northern Genetics Service laboratory.

**Validation:**

The testing protocol was validated in confirming the findings of the research study described below.

**Analytic sensitivity:**

The analytic sensitivity and specificity are close to 100% for the techniques used.

**Clinical validity:**

A study investigating the mutational spectrum of *CYLD *and genotype-phenotype correlation^8^ tested a total of 47 samples from 25 families exhibiting BSS, MFT or FC phenotypes. Of these families, 13 had clinical features of BSS, three of FC and nine of MFT. Mutations in *CYLD* were present in 11/13 with BSS (85%), 3/3 with FC (100%) and 4/9 families with MFT (44%). This gave an overall mutation detection rate of 18/25 (72%) in these families.

## Clinical Utility

The genetic predisposition to these tumours is rare. Although cylindromas carry a significant burden of disease, they are usually not life threatening, which may explain why there are few studies looking at the benefits of genetic testing in affected or at risk individuals. Anecdotal evidence is described in one study of 26 affected patients.^2^ In this study, issues relating to genetic counselling which are relevant to those undergoing genetic testing for *CYLD* mutations are explored. When a pathogenic mutation in *CYLD *is identified in a patient who meets the criteria for testing, they have the advantage of knowing their diagnosis is confirmed, and can use the information for their benefit. Patients who present with multiple skin appendage tumours but are not known to have a family history may not have considered that their tumours could represent an underlying genetic cause. The knowledge that this is so can help them anticipate the fact that further tumours may develop and prepare for necessary treatment. Testing is also facilitated for family members should they develop tumours. Individuals with a family history but no tumours themselves are aware they are at 50% risk of having a *CYLD *mutation. Having the test on a presymptomatic basis can reassure patients if they receive a negative result, and allow them to make decisions about the future if they know the result is positive. Although prenatal diagnostic testing is not offered for this condition, some patients with a *CYLD* mutation may choose to use the information to influence family planning decisions. Patients with a mutation may choose to be entered into appropriate clinical trials when the opportunity arises.

## Links

UKGTN Homepage: http://ukgtn.nhs.uk/

UKGTN Test Dossier: http://ukgtn.nhs.uk/find-a-test/gene-dossiers/

UKGTN Testing Criteria: http://ukgtn.nhs.uk/resources/testing-criteria/

## Competing Interests

The authors have declared that no competing interests exist.
